# Backward Compatible Identity-Based Encryption

**DOI:** 10.3390/s23094181

**Published:** 2023-04-22

**Authors:** Jongkil Kim

**Affiliations:** Department of Cyber Security, Ewha Womans University, Seoul 03760, Republic of Korea; jongkil@ewha.ac.kr

**Keywords:** identity-based encryption, public key encryption, revocation, IoT network security, cloud security

## Abstract

In this paper, we present a new identity-based encryption (IBE) system that is named Backward Compatible Identity-based Encryption (BC-IBE). Our BC-IBE is proposed to solve the problem caused by the out-of-synchronization between users’ private keys and ciphertexts. Encryption systems such as revocable IBE or revocable Attribute-based Encryption (ABE) often require updating private keys to revoke users after a certain time period. However, in those schemes, an updated key can be used to decrypt the ciphertexts created only during the current time period. Once the key is updated and the previous keys are removed, the user, the owner of the updated key, will lose access to the past ciphertexts. In our paper, we propose BC-IBE that supports backward compatibility, to solve this problem. In our proposed system, user’s private keys and ciphertexts can be updated periodically with time tags, and these processes can be used to revoke users who do not receive an updated key as the other revocable encryption does. However, in our proposed system, a private key newly issued to a user is backward compatible. This means that it decrypts not only the ciphertexts at the present time period but also all past ciphertexts. This implies that our proposed scheme guarantees the decryption of all encrypted data even if they are not synchronized. Compared to the existing revocable identity-based encryption system, our proposed BC-IBE has the advantage of simplifying key management and securely delegating ciphertext updates. Our proposed scheme only requires a single backward-compatible private key to decrypt all past ciphertexts created. Moreover, the ciphertext update process in our proposed scheme does not require any special privileges and does not require decryption. This means that this process can be securely delegated to a third-party server, such as a cloud server, and it prevents the potential leakage of secrets. For those reasons, BC-IBE is suitable for a system where users are more dynamic, such as the Internet-of-Things (IoT) network, or a system that regularly updates the data, like cloud data storage. In this paper, we provide the construction of BC-IBE and prove its formal security.

## 1. Introduction

The Internet of Things (IoT) network is a network where a number of heterogeneous devices are connected to each other and exchange various types of data. As the data in an IoT network is often private, the security of the transmitted data in the IoT network is considered important. Public key encryption is the most widely used cryptographic system to control access to the IoT network in security protocols like Transport Layer Security (TLS), Datagram TLS, and Constraint Application Protocol (CoAP) because it does not require any pre-shared secret.

In such public key encryption systems, the authentication of devices is essential because the devices must check the identities of the corresponding party before encrypting a secret using its public key. Otherwise, the adversary can easily disguise as the other and hijack the data in the middle via an attack like Man-in-the-middle (MITM) attack [1,2]. Particularly, in a public key encryption system, a public key is transmitted through a non-secure channel. Therefore, a sender needs to check if the recipient’s public key is matched with its identity so that it is truly from the same recipient who the sender intends to communicate with.

Public Key Infrastructure (PKI) is a widely used system for authentication and preventing this type of attack. A PKI system enables devices to authenticate if the recipient’s identity and public key are matched before the sender sends any private data to the receiver. This conventional system implements the authentication process by issuing and maintaining certificates. Each certificate consists of identity, public keys and digital signatures so that it can be used to verify if the public key in the certificate belongs to the certificate owner via digital signatures. Unfortunately, maintaining certificates causes a significant burden to the system. It requires verifying multiple signatures for authentication and having to exchange large-sized certificates in addition to the cost of maintaining the certificate chain. Those are considered too large for the resource-constrained network such as IoT systems.

Identity-based encryption (IBE) [3,4,5] was introduced to manage access control based on the user’s identity. In IBE, a sender encrypts a message with a public key that is associated with a receiver’s identity. Furthermore, the receiver can decrypt ciphertexts with its private key that corresponds to the public key. The recipient’s public key is not needed to be authenticated as it is already associated with its identity. Therefore, it reduces the burden of managing certificates that the IoT system wants to avoid due to its cost. Although an IBE system can bring a huge benefit to access control in an IoT system where a complex authentication scheme is needed due to its heterogeneity and scale, there are other properties that should be considered in an authentication. One of the properties that must be considered is dynamic access control.

In an IoT system, a user can join and be revoked from the system while the system is operating. One of the key features of IoT networks is sharing resources. A user can use multiple devices to share storage, as each individual device has relatively little storage. Cloud storage services such as Microsoft Onedrive and Google Drive are often used to share users’ data across multiple devices, from desktop PCs to mobile phones or tablet PCs. However, those mobile devices can easily be lost and also out-of-synchronized by the carelessness of the user. This will put users’ privacy at significant risk as those incidents will make a user’s private key and data leak or become inaccessible from time to time.

In a traditional PKI system, a user (or its public key associated with the compromised private key) can be removed from its certificate chain by adding its certificate to a revocation list. However, in the IBE system, which does not use any certificates, the revocation is not straightforward. To support the revocation in IBE systems, Revocable IBE (RIBE) [6,7,8,9,10] was introduced to revoke a user when its private key is compromised.

RIBE uses a key-update mechanism. In RIBE, for a specific time slot, a user’s long-term private key, for which the associated identity does not belong to the revocation list, can be used to compute the decryption key that can be used to decrypt the ciphertext encrypted in that time slot. Therefore, in the RIBE, one who does not have the decryption key in a specific time slot T is revoked. In RIBE, the decryption key cannot be used for the other time slots, particularly ciphertexts for the previous time slots. This may cause some compatibility problems in a practical system. For example, in the heterogeneous IoT network, some nodes are not synchronized properly and encrypt the data using the just previous time slot. The receiver cannot decrypt the data as it has already updated its key. Moreover, in cloud storage, the data is normally synchronized but not always. Some data, for example, stored in local storage, may not be synchronized properly. In this scenario, the data that are encrypted cannot be decrypted after the key update.

In this paper, we solve this problem, which we called the backward compatibility problem, by providing a new encryption system based on IBE that is named Backward Compatible IBE (BC-IBE). Trivial solutions for this problem are (1) keeping all previous decryption keys for backward compatibility or (2) decrypting and re-encrypting all ciphertexts created in the past time slots. However, the former requires more resources in secure memory, which is considered expensive. The latter requires a large overhead and the potential leakage of the secret as it causes decryption and re-encryption.

In our solution, BC-IBE allows a user to keep only a single key for the present time slot, but this key can be used to decrypt not only the ciphertexts created for the present time slot where the current key is issued but also all past ciphertexts previously created. Moreover, at the same time, in our proposed BC-IBE, ciphertexts can be updated for a new time slot without decryption, so that it reduces the overhead caused by decryption and re-encryption.

### 1.1. Our Contribution

In this paper, we propose a new IBE encryption system, which is named backward compatible identity-based encryption (BC-IBE). Our proposed scheme provides the revocation of an expired private key via the update of ciphertexts and the backward compatibility of an updated private key. The details are as follows:

1. Backward compatibility: In our proposed scheme, a private key updated for time T can decrypt all the ciphertexts created at the time T and all the previous ciphertexts created before the time T. One of the most trivial ways to achieve this is to keep all previous keys in secure storage. However, in that system, the user needs to maintain multiple keys to decrypt all past ciphertexts, which consumes large amounts of secure memory space. In our proposed scheme, the user only needs to maintain a single key at all times, but this key can be used to decrypt all past ciphertexts that were encrypted at previous time slots. At the same time, the same key can decrypt the ciphertext generated for the present time slot, too.
2. Revocation: In our proposed scheme, a private key is efficiently revoked by updating ciphertexts in the past time slots. That means that for all ciphertexts of the time T, all previous keys issued before the time T cannot be used to decrypt the ciphertexts at time T. Therefore, all past keys are revoked in the system.
3. Updating ciphertexts: In our BC-IBE, ciphertexts can be updated to revoke the past keys without decryption or re-encryption. Hence, the data owner does not need to download all ciphertexts and decrypt and re-encrypt them for the update. Moreover, this process does not involve any secret parameters. It can be easily delegated to a third party, such as a cloud service provider. Our scheme allows the updating of ciphertexts. This will be helpful for the overall scheme as (1) the scheme does not leak any information to the server while there is no secret involved in updating ciphertexts, (2) the server does not maintain the secret key for the ciphertext update and does not need any connection to be maintained to receive it.

We compare the above three properties of our BC-IBE scheme to the other encryption systems: identity-based encryption (IBE), revocable identity-based encryption (RIBE) and aggregate identity based encryption (AIBE) using Table 1. The plain IBE schemes [3,4,5,11,12] do not support revocation. As there are no states or separated time periods, backward compatibility is not applicable in the plain IBE. Due to the same reason, plain IBE does not support re-encrypting ciphertexts. RIBE [7,9,10,13,14] is a scheme for revoking invalid users only at a specific time slot. All ciphertexts in the past time slot cannot be decrypted using the decryption key at the present time slot, as each decryption key is a short-term key only for the current time slot. Therefore, it does not support the backward compatibility of a private key. The ciphertexts of the past time periods are accessible via decryption and re-encryption for the current time period.

AIBE [15] may be a suitable scheme for backward compatibility, as it can aggregate all past keys and the current key into a single one. However, it is not a scheme for revocation. Therefore, it does not provide revocation and re-encryption.

To summarize, our proposed scheme has the following contribution:

- We define the backward compatibility property of identity-based encryption and construct the first scheme that satisfies the backward compatibility property.
- Together with the backward compatibility property, our scheme supports the update of the ciphertexts. In our scheme, previous ciphertexts can be updated for the newly updated key without decryption, and they cannot be decrypted by all previously issued keys.
- As the encryption system we proposed is new, we newly develop the definition of BC-IBE, suggest its security model, and provide the security proof of the proposed scheme.

### 1.2. Paper Organization

The rest of this paper is organized as follows: in Section 1, we provide the introduction and contribution to explaining the motivation of our research. Section 2 and Section 3 are for related work and preliminary explanations of the important literature for our work, respectively. In Section 4, the detailed method and the technical overview of our BC-IBE are explained. In Section 5, we present our construction and its formal security analysis. In Section 6. We discuss a potential threat and conclude our paper.

## 2. Related Works

The concept of identity-based encryption was proposed by Shamir [3]. It enables users to encrypt a message using their identities, such as e-mail, mobile numbers, and account numbers. Therefore, it reduces a lot of the burden needed to authenticate the receivers. The first practical IBE scheme was proposed by Boneh and Franklin [4] in a bilinear pairing group. Furthermore, it became an active research topic in public-key cryptography. Multiple IBE schemes [5,11,12] which improved security, were introduced, including adaptive security.

Revocable identity-based encryption (RIBE) is a system that improves an IBE scheme to support efficient revocation. As a user’s private key can be compromised by an adversary, revocation is practically needed. The first practical RIBE scheme were introduced by Boldyreva et al. [13] using the complete subtree (CS) method, which achieves logarithm revocation complexity. Their scheme revokes a user’s access by broadcasting an update key. The revoked users cannot compute a decryption key from the key update, so they are revoked. More schemes that achieve better security [14,16] or are based on different revocation methods [17] (e.g., the subset difference method) were introduced. Some RIBE systems are resistant to decryption key leakage. They are called decryption key exposure resistance (DKER) RIBE schemes [7,9,10]. Moreover, the generic constructions for RIBE from IBE was introduced by [6,8]. A few RIBE schemes also [18,19,20] use a third-party server to update the ciphertexts, similar to our scheme but without supporting backwards compatibility, and it also needs a key for the server to revoke users.

RIBE revokes users by updating decryption keys, but our scheme basically revokes users using the ciphertext and the status. This approach is more widely used in revocable attribute-based encryption (RABE) [21,22,23], in which each user has a private key that is associated with its attributes, and the decryption is allowed when its attributes satisfy a specific function (e.g., Boolean function). In those schemes, ciphertexts are re-encrypted or updated to revoke attributes to supporting dynamic credentials. The decryption key in RIBE can be used to decrypt the ciphertexts created within the same time slots. That is the main difference from our BC-IBE. In RIBE, the past ciphertexts can be updated only via decryption using the old keys, then it needs to be re-encrypted for the present time slot.

Improving the efficiency of those RIBE is still an ongoing problem. Lee et al. [24] and Yinxia et al. [25] presented RIBE schemes with a short key and a short ciphertext. More recently, Keita et al. [26] proposed a RIBE scheme that reduces the size of public keys. However, those schemes focus on reducing the keys in the current time period and do not support backward compatibility. This means their scheme still needs a list of private keys for backward compatibility.

## 3. Preliminaries

### 3.1. Bilinear Pairing

Let’s set G as a group generator that takes a security parameter λ as input and outputs a description of a bilinear group G. For our purposes, we will have G output (*p*, G, $G_{T}$, *e*) where *p* is a prime, G and $G_{T}$ are cyclic groups of order *p*, and $e:G\times G\to G_{T}$ is an efficiently computable non-degenerate bilinear map. We assume that the group operations in G and $G_{T}$ as well as the bilinear map *e* are efficiently computable in polynomial time with respect to λ and that the group descriptions of G and $G_{T}$ include generators of the respective cyclic groups.

### 3.2. Assumption

**Assumption** **1.** The q Decision Bilinear Diffie-Hellman Inversion (q-DBDHI) Assumption [15]. Let G and $G_{T}$ be groups of order p with a bilinear map $e:G\times G\to G_{T}$, and let g be a generator for G. Set $\alpha \overset{R}{\leftarrow }Z_{p}^{*}$ and $b\overset{R}{\leftarrow }\left\{0,1\right\}$. If b=0, set $T\leftarrow e{\left(g,g\right)}^{1/\alpha }$; otherwise, set $T\overset{R}{\leftarrow }G_{T}$. Output $\{g^{\alpha^{i}}:i\in \left[q\right]\}\;\mathrm{and}\;T$. The problem is to guess b.

We define the advantage of the adversary of A to guess *b* correctly as follows:

$$Adv_{A,m}^{q-DBDHI}\left(\lambda \right)=|\mathrm{Pr}\left[b=b^{\prime }\right]-\frac{1}{2}|.$$

### 3.3. Definitions

We write the formal definition of IBE using the syntax of [15].

**Definition** **1** (Identity-Based Encryption (IBE)). *The identity-based encryption for a set of identity spaces $I={\left\{{\left\{0,1\right\}}^{n}\right\}}_{n\in N}$ and a message space M consists of the following PPT algorithm (Setup, KeyGen, Enc, Dec):*

- *Setup (1${}^{\lambda }$, 1${}^{n}$) →(pk,msk): takes as input the security parameter λ, the identity length n. It outputs the public parameters pk and the master secret key msk*.
- *KeyGen* *(msk, id) $\to ({\mathrm{sk}}_{\mathrm{id}})$: takes as input the master secret key msk and the identity id∈I. It outputs a private key ${\mathrm{sk}}_{\mathrm{id}}$.*
- *
Enc* *(m, id, pk) $\to ({\mathrm{ct}}_{\mathrm{id}})$: takes as input a message m∈M, an identity id∈I, and the public parameters* *pk* *. It outputs a ciphertext ${\mathrm{ct}}_{\mathrm{id}}$.*
- *
Dec* *(${\mathrm{sk}}_{\mathrm{id}},{\mathrm{ct}}_{\mathrm{id}}$) →(m/⊥): takes as input a private key ${\mathrm{sk}}_{\mathrm{id}}$, a ciphertext ${\mathrm{ct}}_{\mathrm{id}}$. It outputs a message m or aborts.*

**Correctness.** An IBE scheme is correct if the following holds: for all λ,n∈N, let $\left(\mathrm{pk},\mathrm{msk}\right)\leftarrow \mathrm{Setup}\left(1^{\lambda },1^{n}\right),\;\;{\mathrm{sk}}_{\mathrm{id}}\leftarrow \mathrm{KeyGen}\left(\mathrm{msk},\mathrm{id}\right)$ for $\mathrm{id}\in {\left\{0,1\right\}}^{n}$, and ${\mathrm{ct}}_{\mathrm{id}}\leftarrow \mathrm{Enc}\left(m,\mathrm{mpk},\mathrm{id}\right)$ for m∈M. Furthermore, $m\leftarrow \mathrm{Dec}({\mathrm{sk}}_{\mathrm{id}},{\mathrm{ct}}_{\mathrm{id}})$.

The aggregating secret keys property, introduced in ref. [15], allows aggregating multiple private keys in an identity-based encryption scheme into a single compact key. They provide the definition of Aggregate Identity-Based Encryption(AIBE) by adding two extra algorithms to the definition of IBE as described in the following definition:

**Definition** **2** (Aggregate Identity-Based Encryption (AIBE)). *In addition to the algorithms (Setup, KeyGen, Enc, Dec) that forms an IBE scheme, the aggregating secret keys property requires the following two PPT algorithms (KeyAgg, AggDec) to support secret key aggregation in AIBE.*

1. *KeyAgg* *(* *sk* *${}_{1},\ldots {\mathrm{sk}}_{\ell }$) $\to (\hat{\mathrm{sk}}$) takes as input a sequence of secret keys $\left\{{\mathrm{sk}}_{i}\right\}$ for i∈[ℓ] (for some ℓ>1). It outputs an aggregated $\hat{\mathrm{sk}}$.*
2. *AggDec* *($\hat{\mathrm{sk}},({\mathrm{id}}_{1},\ldots {\mathrm{id}}_{\ell }$),* *ct* *, j) →(m/⊥) takes as input an aggregated private key $\hat{\mathrm{sk}}$, a list of identities $\left\{{\mathrm{id}}_{i}\;|i\in \left[\ell \right]\right\}$, a ciphertext ct and the index j∈[ℓ] that denotes the identity utilized to create* *ct* *. It outputs a message m or aborts.*

**Correctness.** An IBE scheme with aggregating secret keys is correct if the following holds: for all λ,n∈N, let $\left(\mathrm{pk},\mathrm{msk}\right)\leftarrow \mathrm{Setup}\left(1^{\lambda },1^{n}\right),$ identities ${\mathrm{id}}_{i}\in {\left\{0,1\right\}}^{n}$ for i∈[ℓ], every secret key ${\mathrm{sk}}_{i}\leftarrow \mathrm{KeyGen}\left(\mathrm{msk},{\mathrm{id}}_{i}\right)$ for i∈[ℓ], an aggregated secret key $\hat{\mathrm{sk}}\leftarrow \mathrm{KeyAgg}\left({\mathrm{sk}}_{1},\ldots ,{\mathrm{sk}}_{\ell }\right)$ and a ciphertext ct $\leftarrow \mathrm{Enc}(\mathrm{mpk},{\mathrm{id}}_{i},m)$. Furthermore, $m\leftarrow \mathrm{AggDec}(\hat{\mathrm{sk}},\left({\mathrm{id}}_{1},\ldots ,{\mathrm{id}}_{\ell }\right),\mathrm{ct},j)$ for all j∈[ℓ], every message m←M.

**Definition** **3** (Security of IBE [15]). *The security of IBE scheme is defined as follows:*

- *Setup: The challenger runs Setup($1^{\lambda }$, $1^{n}$) to obtain a public key pk. It gives A the public key pk*.
- ***Phase I**: The adversary A requests ${\mathrm{sk}}_{{\mathrm{id}}_{i}}$ for $i\in \{1,\ldots ,q_{1}\}$.*
- ***Challenge**: If **Phase I** is over, the adversary*A *sends messages* $m_{0}$ *and* $m_{1}$ *with the challenge identity* id* *to the challenger where* id* *was not queried in **Phase I**. Furthermore, the challenger chooses a random binary β and runs Enc algorithm to calculate* ${\mathrm{ct}}_{\mathrm{id}*}$ = Enc($m_{\beta }$, id*, pk) and returns (${\mathrm{ct}}_{\mathrm{id}*}$) to A.
- **Phase II**: The adversary A continues to requests private keys ${\mathrm{sk}}_{{\mathrm{id}}_{i}}$ for $i\in \left[q\right]\backslash \left[q_{1}\right]$. For every pair ${\mathrm{id}}_{i}$ such that ${\mathrm{id}}_{i}\neq \mathrm{id}*$, it returns ${\mathrm{sk}}_{{\mathrm{id}}_{i}}$ to the adversary.
- ***Guess*** *: Finally, the adversary A outputs a guess $b^{\prime }\in \left\{0,1\right\}$ and wins the game if $b=b^{\prime }$.*

We define the advantage of the adversary A to win in the game as following:

$${\mathrm{AdvIBE}}_{A,n}\left(\lambda \right)=|\mathrm{Pr}\left[b=b^{\prime }\right]-\frac{1}{2}|.$$

Static security is a weaker notion of security. In static security, the adversary lets the challenger know all the identities to be queried and the challenge identity id* before Setup. We use *AdvIBE*${}_{A,n}^{Static}\left(\lambda \right)$ to denote the static security of IBE.

## 4. Our Method

**Figure 1:**
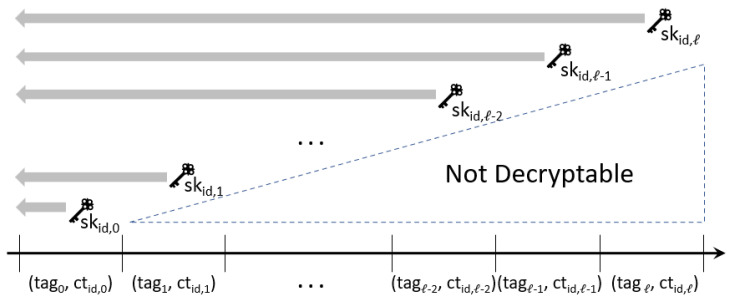
Backward Compatible Identity-Based Encryption (BC-IBE).

The objective of our scheme is to build a more practical revocation system that supports ciphertext update and backward compatibility of the private key. Figure 1 depicts the backward compatibility that our scheme pursues. In the figure, tag${}_{i}$ implies a tag allocated for the *i*th time slot. The private key sk${}_{\mathrm{id},i}$ of an identity id for the time slot *i* can decrypt the ciphertext for the current time slot, ct${}_{\mathrm{id},i}$ and all past ciphertexts, ct${}_{\mathrm{id},j}$ for j<i. However, it cannot decrypt ciphertexts for future time slots.

Our scheme is using a polynomial function to control access. Using a polynomial function is more popular for the variant of IBE, which is Identity-Based Broadcast Encryption (IBBE) [27,28,29]. IBBE is designed to share a single ciphertext for multiple users for broadcasting. As multiple identities are engaged in the encryption and decryption processes, it uses a polynomial function to handle this. In particular, in those schemes, the roots of a polynomial function are identities so it can be used to implement OR gates in the scheme.

In our scheme, a polynomial function is used differently. It has as its roots the identity of the recipient and tags that are uniquely allocated for time slots. So, one who can cancel out a polynomial function embedded in a ciphertext entirely using its private key that is associated with the identity and the tags can decrypt the ciphertext. It means that the polynomial function works in our scheme as AND gates.

For example, let *a*, $t_{1}$ and $t_{2}$ be the identity and the tags used for Alice, respectively. The ciphertext is constructed by using a polynomial function $P\left(a,t_{1},t_{2}\right)=\left(x-a\right)\left(x-t_{1}\right)\left(x-t_{2}\right)$ (e.g., $g^{r\cdot P(a,t_{1},t_{2})}$ where *g* is a group generator and *r* is a randomization parameter.) and Alice has a private key computed based on $1/P(a,t_{1},t_{2})$ (e.g., $g^{1/P(a,t_{1},t_{2})}$) at the second time slot. Therefore, Alice can decrypt the ciphertext using a pairing computation (e.g., $e\left(g^{r\cdot P(a,t_{1},t_{2})},g^{1/P(a,t_{1},t_{2})}\right)=e{\left(g,g\right)}^{r}$) using her key. Therefore, decryption is possible only for one who has a private key based on the inverse of the polynomial function given in the ciphertext.

For the revocation, we observed that, although a polynomial function is set in a ciphertext, it can be updated to become more restrictive. Using the previous example, if the following is given

$$(C=m\cdot e{\left(g,g\right)}^{r},C_{2}=g^{r\cdot P\left(a,t_{1},t_{2}\right)x^{2}},C_{1}=g^{r\cdot P(a,t_{1},t_{2})x},C_{0}=g^{r\cdot P(a,t_{1},t_{2})}).$$

To update the ciphertext with $t_{3}$, the tag for the third time slot is given, and one can update the ciphertext as

$$C^{\prime }=m\cdot e{\left(g,g\right)}^{r},C_{2}^{\prime }=R,\;\;C_{1}^{\prime }=C_{2}\cdot C_{1}^{-t_{3}}=g^{r\cdot P\left(a,t_{1},t_{2}\right)\left(x-t_{3}\right)x}=g^{r\cdot P(a,t_{1},t_{2},t_{3})x},$$

$$C_{0}^{\prime }=C_{1}\cdot C_{0}^{-t_{3}}=g^{rP\left(a,t_{1},t_{2}\right)\left(x-t_{3}\right)}=g^{r\cdot P(a,t_{1},t_{2},t_{3})}$$

where *R* is a random value and $P\left(a,t_{1},t_{2},t_{3}\right)=\left(x-a\right)\left(x-t_{1}\right)\left(x-t_{2}\right)\left(x-t_{3}\right)$. This can be completed without any other secret parameter and does not need decryption. Moreover, once the new ciphertext is updated successfully, it cannot go back.

For the updated ciphertext, all previous keys such as $g^{1/P(a,t_{1},t_{2})}$ are no longer valid as they cannot completely divide the polynomial function given in the ciphertext due to $\left(x-t_{3}\right)$. Therefore, they are revoked. However, the newly generated key at the time $t_{3}$ can be used for all previous ciphertexts by executing the update process locally. Therefore, this method can be used to guarantee backward compatibility.

To prove the security of our scheme, we utilize aggregate identity-based encryption (AIBE). AIBE was introduced by Goyal and Vaikuntanathan [15]. Their scheme is used for a system that maintains multiple private keys. It uses the key accumulate algorithm, called DPP, from Delerablée, Paillier and Pointcheval [30,31] to aggregate multiple keys such as ${\left\{g^{1/(x-a_{i})},a_{i}\right\}}_{i\in [\ell ]}$ into a single key $g^{1/\left(x-a_{1}\right)\left(x-a_{2}\right)\cdots \left(x-a_{\ell }\right)}$. Our scheme does not need key aggregation as a private key can be updated directly by a key-update key. However, it is still useful to prove the security of BC-IBE, as we can query the private keys and use them to form a key-update key without knowing the master secret.

Before we present our main construction, we provide the formal definitions of our BC-IBE and its security models in the following subsections.

### 4.1. Definition of BC-IBE

Using the notion of identity-based encryption, we provide the definition of Backward Compatible Identity-Based Encryption (BC-IBE). Our BC-IBE consists of seven algorithms, Setup, UpdateKeyGen, KeyGen, KeyUpdate, Enc, EncUpdate, and Dec, as defined below:

- Setup($1^{\lambda }$, $1^{n_{1}}$, $1^{n_{2}}$, $1^{n_{3}}$) →(pk,msk): takes as input the security parameter λ, the identity length $n_{1}$, the tag length $n_{2}$ and the maximum number of updates $n_{3}$. It outputs a public key pk and a master secret key msk.
- UpdateKeyGen($\left({\mathrm{tag}}_{1},\ldots ,{\mathrm{tag}}_{\ell }\right),\mathrm{id},\mathrm{msk})\to \left({\mathrm{usk}}_{\mathrm{id},\ell }\right)$: takes as input a sequence of tags ${\mathrm{tag}}_{i}\in {\left\{0,1\right\}}^{n_{2}}$ for i∈[ℓ], an identity id $\in {\left\{0,1\right\}}^{n_{1}}$ and the secret key msk. It outputs a key-update key usk${}_{\mathrm{id},\ell }$.
- KeyGen(id, msk) $\to {\mathrm{sk}}_{\mathrm{id},0}$: takes as input an identity id and the master secret key msk. It outputs a private key ${\mathrm{sk}}_{\mathrm{id},0}$.
- KeyUpdate(${\mathrm{sk}}_{\mathrm{id},j-1}$, usk${}_{\mathrm{id},j})\to {\mathrm{sk}}_{\mathrm{id},j}$: takes as input the private key ${\mathrm{sk}}_{\mathrm{id},j-1}$ and the update key ${\mathrm{usk}}_{\mathrm{id},j}$ for $j\leq n_{3}$. It outputs a private key ${\mathrm{sk}}_{\mathrm{id},j}$.
- Enc(*m*, id, pk) $\to {\mathrm{ct}}_{0}$: takes as input a message m∈M, an identity $\mathrm{id}\in {\left\{0,1\right\}}^{n_{2}}$, and a public key pk. It outputs a ciphertext ${\mathrm{ct}}_{0}$.
- EncUpdate(pk, ${\mathrm{ct}}_{\mathrm{id},j-1}$, $\mathrm{id},({\mathrm{tag}}_{1},\ldots ,{\mathrm{tag}}_{j}$)) $\to {\mathrm{ct}}_{\mathrm{id},j}$: takes as input the ciphertext ${\mathrm{ct}}_{\mathrm{id},j-1}$, an identity id, a sequence of tags $\left({\mathrm{tag}}_{1},\ldots ,{\mathrm{tag}}_{j}\right)$. The algorithm outputs the updated ciphertext ${\mathrm{ct}}_{\mathrm{id},j}$.
- Dec(${\mathrm{sk}}_{\mathrm{id},i},{\mathrm{ct}}_{\mathrm{id},j}$) →m/⊥: takes as input a private key ${\mathrm{sk}}_{\mathrm{id},i}$, a ciphertext ${\mathrm{ct}}_{\mathrm{id},j}$. It outputs the message *m* or aborts.

**Correctness**. For the correctness of a BC-IBE scheme, the following property must be satisfied: for all $\lambda ,n_{1},n_{2},n_{3}\in N$, let $\left(\mathrm{pk},\mathrm{msk}\right)\leftarrow \mathrm{Setup}\left(1^{\lambda },1^{n_{1}},1^{n_{2}},1^{n_{3}}\right)$, ${\mathrm{sk}}_{\mathrm{id},0}\leftarrow \mathrm{KeyGen}\left(\mathrm{id},\mathrm{msk}\right)$ for every identity $\mathrm{id}\in {\left\{0,1\right\}}^{n_{1}}$ and ${\mathrm{ct}}_{\mathrm{id},0}\leftarrow \mathrm{Enc}\left(m,\mathrm{id},\mathrm{pk}\right)$. $m\leftarrow \mathrm{Dec}({\mathrm{sk}}_{\mathrm{id},0},{\mathrm{ct}}_{\mathrm{id},0}$) for every message *m*.
**Correctness for updated ciphertexts and keys**. For correctness for updated ciphertexts and keys of a BC-IBE scheme, the following property must be satisfied: for all $\lambda ,n_{1},n_{2},n_{3}\in N$, let $\left(\mathrm{pk},\mathrm{msk}\right)\leftarrow \mathrm{Setup}\left(1^{\lambda },1^{n_{1}},1^{n_{2}},1^{n_{3}}\right)$, ${\mathrm{sk}}_{\mathrm{id},0}\leftarrow \mathrm{KeyGen}\left(\mathrm{id},\mathrm{msk}\right)$ for every identity $\mathrm{id}\in {\left\{0,1\right\}}^{n_{1}}$. For the update keys, let ${\mathrm{usk}}_{\mathrm{id},i}\leftarrow \mathrm{Update KeyGen}\left(\left({\mathrm{tag}}_{1},\ldots ,{\mathrm{tag}}_{i}\right),\mathrm{id},\mathrm{msk}\right)$ with tags ${\mathrm{tag}}_{1},\ldots {\mathrm{tag}}_{i}\in {\left\{0,1\right\}}^{n_{2}}$ for any $i\leq n_{3}$ and sk${}_{\mathrm{id},i}\leftarrow \mathrm{KeyUpdate}\left({\mathrm{sk}}_{\mathrm{id},i-1},{\mathrm{usk}}_{\mathrm{id},i}\right)$ that is repeatedly computed. Furthermore, let ${\mathrm{ct}}_{\mathrm{id},0}\leftarrow \mathrm{Enc}\left(m,\mathrm{id},\mathrm{pk}\right)$ and ${\mathrm{ct}}_{\mathrm{id},j}\leftarrow \mathrm{EncUpdate}(\mathrm{pk},{\mathrm{ct}}_{\mathrm{id},j-1},\mathrm{id},({\mathrm{tag}}_{1},$$...,{\mathrm{tag}}_{j}))$ for $j\leq n_{3}$. Furthemore, $m\leftarrow \mathrm{Dec}({\mathrm{sk}}_{\mathrm{id},i},{\mathrm{ct}}_{\mathrm{id},j}$) for all j≤i for every message *m*.

### 4.2. Security of BC-IBBE

We define the security of our BC-IBE scheme as follows:

- **Setup**: The challenger runs Setup($1^{\lambda }$, $1^{n_{1}}$, $1^{n_{2}}$, $1^{n_{3}}$) to obtain a public key pk. It gives A the public key pk.
- **Phase I**: The adversary A issues the following queries to the challenger:
  1. For $i\in [q_{1}]$, it requests ${\mathrm{sk}}_{{\mathrm{id}}_{i},0}$.
  2. For $i\in [q_{1}]$, the challenger sets ${\mathrm{tag}}_{1},\;\ldots ,\;{\mathrm{tag}}_{k}\in {\left\{0,1\right\}}^{n_{2}}$ for $k\in [n_{3}]$ and it requests ${\mathrm{usk}}_{{\mathrm{id}}_{i},k}$ for $\left({\mathrm{id}}_{i},{\left\{{\mathrm{tag}}_{j}\right\}}_{j\in [k]}\right)$.
  For each query, the challenger returns the resulting key to the adversary.
- **Challenge**: When **Phase I** is over, the adversary A sends messages $m_{0}$ and $m_{1}$ with a challenge of identity id* and tags, $\left({\mathrm{tag}}_{1}^{*},\ldots ,{\mathrm{tag}}_{\ell }^{*}\right)$ in that the pair $\left(\mathrm{id}*,{\mathrm{tag}}_{\ell }^{*}\right)$ has never been queried together in **Phase I** to the challenger. The challenger chooses a random binary β and runs the Enc algorithm to compute ${\mathrm{ct}}_{\mathrm{id}*,0}$ = Enc($m_{\beta }$, id*, pk), and then it updates the ciphertext ${\mathrm{ct}}_{\mathrm{id}*,0}$ to the ciphertext ${\mathrm{ct}}_{\mathrm{id}*,j}$ using EncUpdate by executing consecutively EncUpdate$\left(\mathrm{pk},{\mathrm{ct}}_{\mathrm{id}*,i-1},\mathrm{id}*,\left({\mathrm{tag}}_{1}^{*},\ldots ,{\mathrm{tag}}_{i}^{*}\right)\right)$ for i∈[ℓ]. The challenger returns (${\mathrm{ct}}_{\mathrm{id}*,\ell }$) to A.
- **Phase II**: The adversary A continues to issue the following queries:
  1. For $i\in \left[q\right]\backslash \left[q_{1}\right]$, it requests ${\mathrm{sk}}_{{\mathrm{id}}_{i},0}$.
  2. For $i\in \left[q\right]\backslash \left[q_{1}\right]$, the challenger sets ${\mathrm{tag}}_{1},\;\ldots ,\;{\mathrm{tag}}_{k}\in {\left\{0,1\right\}}^{n_{2}}$ such that $k\in [n_{3}]$ and it requests ${\mathrm{usk}}_{{\mathrm{id}}_{i},k}$ for $\left({\mathrm{id}}_{i},{\left\{{\mathrm{tag}}_{j}\right\}}_{j\in [k]}\right)$ with the restriction that the challenge pair $\left(\mathrm{id}*,{\mathrm{tag}}_{\ell }^{*}\right)$ cannot be queried.
  For each query, the challenger forwards the resulting key to the adversary.
- **Guess**: Finally, the adversary A outputs a guess $b^{\prime }\in \left\{0,1\right\}$ and wins the game if $b=b^{\prime }$.

We define the advantage of the adversary A to win in the game as follows:

$$\mathrm{AdvBC}-{\mathrm{IBE}}_{A,n}\left(\lambda \right)=|\mathrm{Pr}\left[b=b^{\prime }\right]-\frac{1}{2}|.$$

Static security is the weaker security notion of BC-IBE by adding the step that the adversary lets the challenger know all identities and tags to be queried before Setup (i.e., before requesting any parameters from the challenger). We use *AdvBC*-*IBE*${}_{A,n}^{Static}\left(\lambda \right)$ to denote the advantage of the adversary in the static security model.

## 5. Our Result

### 5.1. Our Construction

Let G(λ) be an algorithm that outputs bilinear group parameters $\langle G,G_{T},e\rangle$, where G and $G_{T}$ are of order *p*, and $e:G\times G\to G_{T}$. Let *g* and e(g,g) be generators of G and $G_{T}$, respectively. With a collision-resistant hash algorithm $H:{\left\{0,1\right\}}^{n_{1}}\times {\left\{0,1\right\}}^{n_{2}}\to Z_{p}$ where $n_{1}$ and $n_{2}$ are the lengths of identities and tags, respectively. Furthermore, $n_{3}$ is the maximum number of updates. The construction of our BC-IBE scheme is as follows:

- Setup$\left(1^{\lambda },1^{n_{1}},1^{n_{2}},1^{n_{3}}\right)\to \left(\mathrm{pk},\mathrm{msk}\right)$ takes the security parameter λ as input and the sizes of identities ($n_{1}$) and tags ($n_{2}$) together with the maximum number of the key updates ($n_{3}$). Furthermore, the algorithm generates the bilinear group $\langle p,G,G_{T},g,e\rangle \leftarrow G\left(1^{\lambda }\right)$. It randomly chooses parameters α and β in $Z_{p}$ and hk $\leftarrow \mathrm{HGen}(1^{\lambda })$ where hk is a parameter for the identity hash algorithm. It sets a public key pk $:=(\mathrm{hk},g,{\left\{g^{\beta^{i}}\right\}}_{i\in [n_{3}]},g^{\alpha })$ and a private key msk :=(hk,α,β).
- UpdateKeyGen($\left({\mathrm{tag}}_{1},\ldots ,{\mathrm{tag}}_{\ell }\right),\mathrm{id},\mathrm{msk})\to \left({\mathrm{usk}}_{\mathrm{id},\ell }\right)$: takes the set of tags $\left({\mathrm{tag}}_{1},\ldots {\mathrm{tag}}_{\ell }\right)$ as inputs, an identity id and the master secret key msk. It sets $h_{\mathrm{id},i}=H\left(\mathrm{hk},\mathrm{id},{\mathrm{tag}}_{i}\right)$ for i∈[ℓ]. It sets ${\mathrm{usk}}_{\mathrm{id},\ell }=g^{\alpha /\left(\left(\beta +h_{\mathrm{id},0}\right)\cdots \left(\beta +h_{\mathrm{id},\ell }\right)\right)-\alpha /\left(\left(\beta +h_{\mathrm{id},0}\right)\cdots \left(\beta +h_{\mathrm{id},\ell -1}\right)\right)}$ where $h_{\mathrm{id},0}=H\left(\mathrm{hk},\mathrm{id},0\right)$ for 1≤ℓ. It outputs ${\mathrm{usk}}_{\mathrm{id},\ell }$.
- KeyGen(id, msk) $\to {\mathrm{sk}}_{\mathrm{id},0}$: The key generation algorithm takes the identity id and the master secret key msk. It then outputs ${\mathrm{sk}}_{\mathrm{id},0}=g^{\alpha /(\beta +h_{\mathrm{id},0})}$ where $h_{\mathrm{id},0}=H\left(\mathrm{hk},\mathrm{id},0\right)$.
- KeyUpdate(${\mathrm{sk}}_{\mathrm{id},\ell -1}$, ${\mathrm{usk}}_{\mathrm{id},\ell }$) $\to {\mathrm{sk}}_{\mathrm{id},\ell }$: The key generation algorithm takes the latest updated key ${\mathrm{usk}}_{\mathrm{id},\ell }$ and the secret key ${\mathrm{sk}}_{\mathrm{id},\ell -1}$ for 1≤ℓ. It updates the secret key
  $${\mathrm{sk}}_{\mathrm{id},\ell }={\mathrm{sk}}_{\mathrm{id},\ell -1}\cdot {\mathrm{usk}}_{\mathrm{id},\ell }.$$
  It should be noted that the above equation results in ${\mathrm{sk}}_{\mathrm{id},\ell }=g^{\alpha /\prod_{i=0}^{\ell }\left(\beta +h_{\mathrm{id},\ell }\right)}$. It returns ${\mathrm{sk}}_{\mathrm{id},\ell }$.
- Enc$\left(m,\mathrm{pk},\mathrm{id}\right)\to {\mathrm{ct}}_{\mathrm{id},0}$: The encryption algorithm takes a message *m*, a public key pk and an identity id. It randomly selects a random value $r\in Z_{p}$ and computes $h_{\mathrm{id},0}=H\left(\mathrm{hk},\mathrm{id},0\right)$. It, then, sets the following as the ciphertext ct:
  $$C_{i}:=g^{r\left(\beta +h_{\mathrm{id},0}\right)\beta^{i}},C_{T}:=m\cdot e{\left(g,g\right)}^{\alpha \cdot r}$$
  It outputs $\mathrm{ct}:=\left({\left\{C_{i}\right\}}_{i=0}^{n_{3}},C_{T}\right)$.
- EncUpdate$\left(\mathrm{pk},{\mathrm{ct}}_{\mathrm{id},\ell -1},\ell ,\mathrm{id},\left({\mathrm{tag}}_{1},\ldots ,{\mathrm{tag}}_{\ell }\right)\right)\to \left({\mathrm{ct}}_{\mathrm{id},\ell }\right)$: The re-encryption algorithm takes a public key pk and a ciphertext ${\mathrm{ct}}_{\mathrm{id},\ell -1}$ and the identity id and a sequence of the tags $\left({\mathrm{tag}}_{1},\ldots ,{\mathrm{tag}}_{\ell }\right)$. It randomly selects a random value $r^{\prime }\in Z_{p}$ and computes $\left(h_{\mathrm{id},0},h_{\mathrm{id},1},\ldots ,h_{\mathrm{id},\ell }\right)=(H\left(\mathrm{hk},\mathrm{id},0\right),H\left(\mathrm{hk},\mathrm{id},{\mathrm{tag}}_{1}\right)$$,\ldots ,H(\mathrm{hk},\mathrm{id},{\mathrm{tag}}_{\ell }))$. It computes $\left(a_{0},a_{1},\ldots ,a_{\ell +1}\right)$ where $a_{j}$ is the coefficient of $x^{j}$ in the polynomial function $\left(x+h_{\mathrm{id},0}\right)\left(x+h_{\mathrm{id},1}\right)\left(x+h_{\mathrm{id},2}\right)\cdots \left(x+h_{\mathrm{id},\ell }\right)$. It computes $C_{i}^{\prime \prime }={\left\{g^{\sum_{j=0}^{\ell +1}a_{j}\cdot \beta^{i+j}}\right\}}_{i=0}^{n_{3}-\ell }$.
  It sets $C_{T}^{\prime }:=C_{T}\cdot e{\left(g,g\right)}^{\alpha r^{\prime }}$. For all $i\in [n_{3}-\ell ]$,
  $$C_{i}^{\prime }:=C_{i+1}\cdot {C_{i}}^{h_{\mathrm{id},i}}\cdot C_{i}^{\prime \prime }$$
  For all *i* such that $n_{3}-\ell <i\leq n_{3}$, it randomly selects $R_{i}\in G$ also sets $C_{i}^{\prime }:=C_{i}\cdot R_{i}$. It outputs the updated ciphertext:
  $${\mathrm{ct}}_{\mathrm{id},\ell }=:\left({\left\{C_{i}^{\prime }\right\}}_{i=0}^{n_{3}},C_{T}^{\prime }\right)$$
- Dec(${\mathrm{sk}}_{\mathrm{id},\ell },{\mathrm{ct}}_{\mathrm{id},j},\left({\mathrm{tag}}_{1},\ldots ,{\mathrm{tag}}_{\ell }\right),j,\mathrm{hk})\to \left(m\right)$: The decryption algorithm takes the secret key ${\mathrm{sk}}_{\mathrm{id},\ell }$ and the ciphertext ${\mathrm{ct}}_{\mathrm{id},j}$ of id and the tags $\left\{{\mathrm{tag}}_{1},\ldots {\mathrm{tag}}_{\ell }\right\}$. Furthermore, we set coefficient $a_{i}$ as follows:
  – If j=ℓ, $a_{0}=1$.
  – If j<ℓ, for all i∈{0,…,ℓ−j}, $a_{i}$ is the coefficients of $x^{i}$ of the polynomial $\left(x+h_{\mathrm{id},j+1}\right)\cdots \left(x+h_{\mathrm{id},\ell }\right)$ where $h_{\mathrm{id},j}=H\left(\mathrm{hk},\mathrm{id},{\mathrm{tag}}_{j}\right)$.
  It, then, parses ${\mathrm{ct}}_{\mathrm{id},j}$ to $C_{0}\ldots C_{n_{3}}$ and $C_{T}$ and computes
  $$m=C_{T}\cdot e{\left(\prod_{i=0}^{\ell -j}{C_{i}}^{a_{i}},{\mathrm{sk}}_{\mathrm{id},\ell }\right)}^{-1}.$$

**Correctness**. By the definition, $C_{0}=g^{r(\beta +h_{\mathrm{id},0})}$, $sk_{\mathrm{id},0}=g^{\alpha /(\beta +h_{\mathrm{id},0})}$ and $C_{T}=m\cdot e{\left(g,g\right)}^{\alpha r}$. Therefore,
  $$\begin{array}{cc} C_{T}\cdot e{\left(C_{0},{\mathrm{sk}}_{\mathrm{id},0}\right)}^{-1} & =m\cdot e{\left(g,g\right)}^{\alpha r}\cdot e{\left(g^{r\cdot (\beta +h_{\mathrm{id},0})},g^{\alpha /(\beta +h_{\mathrm{id},0})}\right)}^{-1}\\ \; & =m\cdot e{\left(g,g\right)}^{\alpha \cdot r}\cdot e{\left(g,g\right)}^{-\alpha \cdot r}\\ \; & =m \end{array}$$
**Correctness for updated ciphertexts and keys**. For j≤ℓ, by the definition, $C_{j}=g^{\hat{r}\prod_{i=0}^{j}\left(\beta +h_{\mathrm{id},i}\right)}$, $sk_{\mathrm{id},\ell }=g^{\alpha /\prod_{i=1}^{\ell }\left(\beta +h_{\mathrm{id},i}\right)}$ and $C_{T}^{\prime }=m\cdot e{\left(g,g\right)}^{\alpha \cdot \hat{r}}$. First, we compute $a_{i}$ that is the coefficient of $x^{i}$ of the polynomial $\left(x+h_{\mathrm{id},j+1}\right)\cdots \left(x+h_{\mathrm{id},\ell }\right)$ for all i∈{0,…,ℓ−j}. Therefore,
  $$\begin{array}{cc} C_{T}^{\prime }\cdot e{\left(\prod_{i=0}^{\ell -j}{C_{i}}^{a_{i}},{\mathrm{sk}}_{\mathrm{id},\ell }\right)}^{-1} & =m\cdot e{\left(g,g\right)}^{\alpha \hat{r}}\cdot e{\left(\prod_{i=0}^{\ell -j}C_{i}^{a_{i}},{\mathrm{sk}}_{\mathrm{id},\ell }\right)}^{-1}\\ \; & =m\cdot e{\left(g,g\right)}^{\alpha \cdot \hat{r}}\cdot e\left(g^{\hat{r}\cdot \left(\prod_{i=0}^{j}\left(\beta +h_{\mathrm{id},i}\right)\right)\left(\sum_{i=0}^{\ell -j}a_{i}\cdot \beta^{i}\right)},g^{\alpha /\prod_{i=0}^{\ell }\left(\beta +h_{\mathrm{id},i}\right)}\right)\\ \; & =m\cdot e{\left(g,g\right)}^{\alpha \cdot \hat{r}}\cdot e\left(g^{\hat{r}\cdot \left(\prod_{i=0}^{\ell }\left(\beta +h_{\mathrm{id},i}\right)\right)},g^{\alpha /\prod_{i=0}^{\ell }\left(\beta +h_{\mathrm{id},i}\right)}\right)\\ \; & =m\cdot e{\left(g,g\right)}^{\alpha \cdot \hat{r}}\cdot e{\left(g,g\right)}^{-\alpha \cdot \hat{r}}\\ \; & =m \end{array}$$

**Theorem** **1.** *Our BC-IBE is static secure under (q-DBDHI) assumption.*

**Proof.**  We will show the security of our BC-IBE using Lemma 1 in our security analysis. It will be proven to be secure by showing the oracles simulating the security of the AIBE from Goyal and Vaikuntanathan are invariant. Therefore, it will have the same security that AIBE has. □

### 5.2. Security Analysis

We utilize Goyal and Vaikuntanathan’s AIBE (see Appendix A) to prove our scheme. Their AIBE scheme is static secure and its static security is proven under *q*-DBDHI assumption in a random oracle model. In our scheme, we also show that our scheme is secure via the security of the AIBE scheme.

To use the AIBE scheme for the security proof of BC-IBE, we define two indistinguishable oracles O${}_{\mathrm{AIBE}}^{0}$ and O${}_{\mathrm{AIBE}}^{1}$ that simulate the static security of AIBE. First, we define O${}_{\mathrm{AIBE}}^{0}$ as follows:

- Setup(1${}^{\lambda }$, 1${}^{n}$, id*) →(mpk): takes as input the security parameter λ, the identity length *n*. It returns the public parameters $\mathrm{mpk}=(\mathrm{hk},{\left\{g^{\beta^{i}}\right\}}_{i\in [n]})$ where hk $\leftarrow \mathrm{HGen}(1^{\lambda })$ is a parameter for the hash algorithm for identities *H* (i.e., $H\left(\mathrm{hk},\mathrm{id}\right)=h_{\mathrm{id}})\in Z_{p}$).
- Query($\mathrm{id}\in {\left\{0,1\right\}}^{n}\backslash \left\{\mathrm{id}*\right\}$) $\to ({\mathrm{sk}}_{\mathrm{id}})$: When a secret key sk${}_{\mathrm{id}}$ for the identity $\mathrm{id}\in {\left\{0,1\right\}}^{n}$ is requested, it returns a private key ${\mathrm{sk}}_{\mathrm{id}}=g^{{\left(\beta +h_{\mathrm{id}}\right)}^{-1}}$ where $h_{\mathrm{id}}=H\left(\mathrm{hk},\mathrm{id}\right)$.
- Challenge$(m_{0},m_{1},\mathrm{id}*,\mathrm{mpk}$) $\to ({\mathrm{ct}}_{\mathrm{id}*})$ takes as input messages $m_{0},m_{1}\in M$, an identity id*∈{0,1}, and the public parameters mpk. It randomly selects b∈{0,1} and outputs the challenge ciphertext
  $${\mathrm{ct}}_{\mathrm{id}*}=({\left\{g^{r\left(\beta +h_{\mathrm{id}*}\right)\beta^{i}}\right\}}_{i=0}^{n},m_{b}\cdot e{\left(g,g\right)}^{r})$$
  where *r* is a randomly selected value in $Z_{p}$.

The oracle O${}_{\mathrm{AIBE}}^{1}$ is defined identically except that $m_{b}$ in ct ${}_{\mathrm{id}*}$ is replaced by a random message in M. It should be noted that the oracles O${}_{\mathrm{AIBE}}^{0}$ and O${}_{\mathrm{AIBE}}^{1}$ are indistinguishable under q−DBDHI assumption in the random oracle model by the static security of Aggregate IBE in [15].

**Lemma** **1.** *Suppose there is a PPT algorithm A that breaks the static security of BC-IBE with non-negligible probability ϵ. Furthermore, we can build an algorithm B that distinguishes between O${}_{\mathrm{AIBE}}^{0}$ and O${}_{\mathrm{AIBE}}^{1}$ using A with ϵ*.

**Proof.**  We are going to prove the static security of our BC-IBE scheme using the indistinguishability between O${}_{\mathrm{AIBE}}^{0}$ and O${}_{\mathrm{AIBE}}^{1}$ as follows:

Before Setup, for the initialization, the challenger sets the identities to be queried and the maximum number of updates $n_{3}$. It also chooses the identity to be challenged, id* and the number of updates to be challenged for id*, which is ${\mathrm{tag}}_{\ell }^{*}$. The algorithm B sets a new identity space that is defined as
  $$\{\{{\mathrm{id}}_{i}\left|\right|{\mathrm{tag}}_{i,j}{\}}_{i\in \left[q\right],j\in \left[n_{3}\right]},\;\;\{\mathrm{id}*\left|\right|{\mathrm{tag}}_{j}{\}}_{j\in [n_{3}]}\}$$
  and the challenge identity $\mathrm{id}*\left|\right|{\mathrm{tag}}_{\ell }^{*}$. B sends them to the oracle (either O${}_{\mathrm{AIBE}}^{0}$ or O${}_{\mathrm{AIBE}}^{1}$) that it works with. The oracle uses this for the initialization by creating $n_{1}+n_{2}$ sized identity space (i.e., ${\left\{0,1\right\}}^{n_{1}+n_{2}}$ where $n_{1}$ and $n_{2}$ are the sizes of the identity and tag spaces, respectively. Through this process, B will break the static security of AIBE by distinguishing between O${}_{\mathrm{AIBE}}^{0}$ and O${}_{\mathrm{AIBE}}^{1}$ using A.
Setup: To create pk for B. B requests a public key to the oracle, it works with. The oracle sends $\left(\mathrm{hk},{\left\{g^{\beta^{i}}\right\}}_{i\in [n]}\right)$ back to B. Furthermore, B randomly selects α and sends $\left(H,{\left\{g^{\beta^{i}}\right\}}_{i\in [n]},g^{\alpha }\right)$ as pk where *H* is a random oracle hashing identities with hk $\leftarrow \mathrm{HGen}(1^{\lambda })$ by concatenating an identity and a tag and taking it as input together with hk. (i.e., *H*(hk, id|| tag)).
Phase I/II: In this stage, A can query three types of queries and B responds it as follows:

1. When A requests ${\mathrm{sk}}_{{\mathrm{id}}_{i},0}$ for i∈[q], B requests the private key for ${\mathrm{id}}_{i}\left|\right|0$ to the oracle. B receives ${\hat{\mathrm{sk}}}_{{\mathrm{id}}_{i}\left|\right|0}$ from the oracle. Furthermore, it sets ${\mathrm{sk}}_{{\mathrm{id}}_{i},0}$ = ${\left({\hat{\mathrm{sk}}}_{{\mathrm{id}}_{i}\left|\right|0}\right)}^{\alpha }$ and returns it to A.
2. When A requests usk ${}_{{\mathrm{id}}_{i},j}$ for ${\mathrm{id}}_{i}\left|\right|{\mathrm{tag}}_{i,j}$ for $i\in \left[q\right],j\in \left[n_{3}\right]$, B requests the a private key for ${\mathrm{id}}_{i}\left|\right|{\mathrm{tag}}_{i,j}$ to the oracle. B receives ${\hat{\mathrm{sk}}}_{{\mathrm{id}}_{i}\left|\right|{\mathrm{tag}}_{i,j}}$ from the oracle it works with. Furthermore, it computes $d_{1}=\mathrm{DPP}\left({\hat{\mathrm{sk}}}_{{\mathrm{id}}_{i}\left|\right|0},{\hat{\mathrm{sk}}}_{{\mathrm{id}}_{i}\left|\right|{\mathrm{tag}}_{i,1}},\ldots ,{\hat{\mathrm{sk}}}_{{\mathrm{id}}_{i}\left|\right|{\mathrm{tag}}_{i,j-1}}\right)$ and $d_{2}=\mathrm{DPP}\left({\hat{\mathrm{sk}}}_{{\mathrm{id}}_{i}\left|\right|0},{\hat{\mathrm{sk}}}_{{\mathrm{id}}_{i}\left|\right|{\mathrm{tag}}_{i,1}},\ldots ,{\hat{\mathrm{sk}}}_{{\mathrm{id}}_{i}\left|\right|{\mathrm{tag}}_{i,j}}\right)$. It sets usk${}_{{\mathrm{id}}_{i},j}={\left(d_{2}\right)}^{\alpha }-{\left(d_{1}\right)}^{\alpha }$ and returns it to A where the function DPP [30,31] is an aggregate algorithm that can aggregate multiple keys such as ${\left\{g^{1/(x+a_{i})},a_{i}\right\}}_{i\in [\ell ]}$ into a single key $g^{1/\left(x+a_{1}\right)\left(x+a_{2}\right)\cdots \left(x+a_{\ell }\right)}$.
3. A also can request sk${}_{\mathrm{id}*\left|\right|0}$ and usk${}_{\mathrm{id}*\left|\right|{\mathrm{tag}}_{i}^{*}}$ for i∈[ℓ−1] and also B can respond in the same way it responds above. They cannot query usk${}_{\mathrm{id}*\left|\right|{\mathrm{tag}}_{\ell }^{*}}$.

Challege: When A requests the challenge ciphertext for id*, $\left({\mathrm{tag}}_{1}^{*},\ldots ,{\mathrm{tag}}_{\ell }^{*}\right)$, B requests the challenge ciphertext of $\mathrm{id}*\left|\right|{\mathrm{tag}}_{\ell }^{*}$ to the oracle that it works with. The oracle will send the challenge ciphertext that is either a ciphertext for $m_{b}$ or a random message following:
  $$\mathrm{ct}=({\left\{g^{r\left(\beta +h_{\mathrm{id}*\left|\right|{\mathrm{tag}}_{\ell }^{*}}\right)\beta^{i}}\right\}}_{i=0}^{n_{3}},T)$$
  where *T* is either $m_{b}\cdot e{\left(g,g\right)}^{r}$ or a random value $R\in G_{T}$ according to the definition of the oracles. To create the challenge cipherttext for A, B requests $\left(h_{\mathrm{id}*\left|\right|0},h_{\mathrm{id}*\left|\right|{\mathrm{tag}}_{1}^{*}},\ldots ,h_{\mathrm{id}*\left|\right|{\mathrm{tag}}_{\ell -1}^{*}}\right)$ to the oracle it works with. It then, computes $a_{0},\ldots ,a_{\ell }$ where $a_{i}$ is a coefficient of $x^{i}$ of $\left(x+h_{\mathrm{id}*\left|\right|0}\right)\left(x+h_{\mathrm{id}*\left|\right|{\mathrm{tag}}_{1}^{*}}\right)\cdots \left(x+h_{\mathrm{id}*\left|\right|{\mathrm{tag}}_{\ell -1}^{*}}\right)$.
  $$\begin{array}{cc} \mathrm{ct} & =(\{R_{1},\ldots ,R_{\ell },{\left\{g^{r\left(\beta +h_{\mathrm{id}*\left|\right|{\mathrm{tag}}_{\ell }^{*}}\right)\left(a_{\ell }\beta^{\ell }+\ldots +a_{1}\beta^{1}+a_{0}\right)\beta^{i}}\right\}}_{i=0}^{n_{3}-\ell }\},T)\\ \; & =(\{R_{1},\ldots ,R_{\ell },{\left\{g^{r\left(\beta +h_{\mathrm{id}*\left|\right|{\mathrm{tag}}_{\ell }^{*}}\right)\left(\beta +h_{\mathrm{id}*\left|\right|0}\right)\left(\beta +h_{\mathrm{id}*\left|\right|{\mathrm{tag}}_{1}^{*}}\right)\cdots \left(\beta +h_{\mathrm{id}*\left|\right|{\mathrm{tag}}_{\ell -1}^{*}}\right)\beta^{i}}\right\}}_{i=0}^{n_{3}-\ell }\},T) \end{array}$$
  where $R_{1},\ldots ,R_{\ell }$ are random values in $Z_{p}$ It, then, returns the challenge chiphertext to A. The last equality of the above equation because, for all i={0,1,…,ℓ}, $a_{i}$ is also the coefficient of $\beta^{i}$ of $\left(\beta +h_{\mathrm{id}*\left|\right|0}\right)\left(\beta +h_{\mathrm{id}*\left|\right|{\mathrm{tag}}_{1}^{*}}\right)\cdots \left(\beta +h_{\mathrm{id}*\left|\right|{\mathrm{tag}}_{\ell -1}^{*}}\right)$ by its definition.
Guess: When B receives the answer from A, it sends back the result to the oracle it works with. As A distinguishes if *T* encrypts a random message or $m_{b}$ with the non-negligible advantage ϵ, B also can use this advantage to distinguish between O${}_{\mathrm{AIBE}}^{0}$ and O${}_{\mathrm{AIBE}}^{1}$. □

### 5.3. Performance Evaluation

In this section, we compare the performance of our scheme with the other revocable identity-based encryption schemes in Table 2 and Table 3. In revocable identity-based encryption schemes [9,17,26], backward compatibility is not supported. Therefore, we assume that they keep all previous keys for backward compatibility for *n* time periods.

As shown in those tables, our scheme has a very short private key (sk) even though it supports backward compatibility. In the existing scheme, the size of those keys increases in not only the number of keys it keeps for backward compatibility, (*n*) but also the total number of users (*ℓ*). Moreover, our key update processes are simple, it always needs a single key for each time period and sk is directly used as a decryption key. In all other schemes, the size of updating keys increases in the total number of users (*ℓ*).

Our scheme has relatively longer ciphertexts as it increases in the maximum number of updates and also the decryption needs *n* exponentiation computations although it only needs one pairing. However, the other RIBE does not support the updates. It needs decryption and re-encryption to update existing ciphertexts. This difference makes the size of the ciphertext larger but we believe that this is the cost of the functional enhancement. In addition, one may consider guaranteeing backward compatibility within reasonable time periods. With a smaller *n*, our scheme outperforms the other RIBE schemes in terms of decryption overhead.

Moreover, the size of the secret keys is often considered an expensive resource as it needs secure memory and is an extra burden for the management. Therefore, our proposed scheme can be used where the restriction of secure memory is severe.

## 6. Discussion and Conclusions

### 6.1. Discussion on Threats on Updating Ciphertexts

In our proposed scheme, the ciphertext update can be conducted by any third party without giving any private key. However, our update only makes the access policy applied to ciphertexts more restrictive because it always needs a newly established update key to be aggregated to a decryption key in addition to the keys already aggregated to the decryption key. Due to this, even one who has a malicious purpose cannot compromise the ciphertext through the update. The other potential threat is one makes ciphertexts inaccessible by updating them to an arbitrary identity. However, this means that the adversary has a writing privilege for the ciphertext because the update process needs to overwrite the previous ciphertext to the updated one. If the adversary already has the writing privilege, the adversary can compromise the availability of ciphertext anyway even without using the update algorithm. For example, it can overwrite ciphertext to any random elements using its privilege. Due to the reasons we explain above, we argue that the threats on updating ciphertext are reasonable and do not increase the attacker’s capability significantly.

### 6.2. Conclusions

In this paper, we introduce a new encryption system, which supports the backward compatibility property. Our proposed scheme allows for a user to keep a single key allocated for the present time slot and this key can decrypt all ciphertexts created at past and present time slots. So, it is backward compatible. In addition, in our proposed scheme, ciphertexts can be updated for the new time slots without decryption. After it is updated, it cannot be decrypted using the past keys. Therefore, it naturally supports revocation. The ciphertext update process does not require any secret parameters in our scheme so that it can be delegated to a third party. We believe that this is helpful for cloud storage and server-aided IoT network where the synchronization of the data in a system matters.

We present our idea by setting a new definition of backward compatible identity-based encryption (BC-IBE) and its security model. Furthermore, we construct an efficient scheme that satisfies the backward compatibility property we previously described. We prove its security using the aggregate identity-based encryption scheme introduced by Goyal and Vaikuntanathan.

We provide an efficient scheme as the size of the private key is constant, but the length of the ciphertext of the proposed scheme increases linearly over the maximum number of updates. It would be interesting to reduce the size of ciphertexts for future work.

## Figures and Tables

**Table 1:** Comparison of BC-IBE to the other encryption systems.

	Backward Compatibility	Revocation	Re-Encryption w/o Decryption
IBE [3,4,5,11,12]	N/A	No	No
RIBE [7,9,10,13,14]	No	Yes	No
AIBE [15]	Maybe	No	No
BC-IBE (Ours)	Yes	Yes	Yes

**Table 2:** Efficiency Comparison with RIBE-parameter sizes. (*n* is the maximum number of backward-compatible ciphertexts. *ℓ* is the maximum number of users.)

	pk	sk with BC	ct
SE [9]	$\left(6+|ID|)\right|G_{p}|$	$2n\left(log\ell \right)|G_{p}|$	$3|G_{p}\left|+\right|G_{T}|$
LLP [17]	$6|G_{N}|$ + $|G_{T}|$	$n\left(log^{1.5}\ell \right)\left|G_{N}\right|$	$4|G_{N}|$
ESW [26]	$7|G_{1}|$ + $11|G_{2}|$ + $|Z_{p}|$	$n\left(5log\ell \right)|G_{2}|$	$4|G_{1}|$ + $|G_{T}|$ + $|Z_{p}|$
Ours	$\left(n+2\right)|G_{p}|$ + $|Z_{p}|$	$|G_{p}|$	$\left(n+1\right)|G_{p}|$ + $|G_{T}|$

**Table 3:** Efficiency Comparison with RIBE - Updating keys and # of pairing in Decryption (r is the number of revoked users. *ℓ* is the maximum number of users.

	Update Key	Decryption Key	# of Pairing in Dec
SE [9]	$\left(2rlog\left(\ell /r\right)\right)|G_{p}|$	$3|G_{p}|$	3 Pairings
LLP [17]	$4r|G_{N}|$	$3|G_{N}|$	3 Pairings + 10 Exp.
ESW [26]	$3rlog\left(\ell /r\right))|G_{2}|$	$6|G_{2}|$	3 Pairings + 2 Exp.
Ours	$r|G_{p}|$	$|G_{p}|$	1 Pairing + r Exp.

## Data Availability

Data sharing not applicable.

## Appendix A. Goyal and Vaikuntanathan’s Aggregate IBE

We provide the construction of Goyal and Vaikuntanathan’s Aggregate IBE [15]. We let the bilinear group $\langle p,G,G_{T},g,e\rangle \leftarrow G\left(1^{\lambda }\right)$ be the bilinear group parameters in prime order *p*.

- Setup$\left(1^{\lambda },1^{n}\right)\to \left(\mathrm{pk},\mathrm{msk}\right)$ takes the security parameter λ as input and the upper bound on the number of aggregations *n*. It randomly samples a value $\beta \in Z_{p}^{*}$ and the public parameters for identity hashing as hk $\leftarrow \mathrm{HGen}(1^{\lambda })$. It sets a public key pk $:=(\mathrm{hk},{\left\{g^{\beta^{i}}\right\}}_{i\in [n]},g^{\alpha })$ and a private key msk :=(hk,β).
- KeyGen(id, msk) $\to {\mathrm{sk}}_{\mathrm{id}}$: The key generation algorithm takes the identity id and the master secret key msk. It hashes the identity as $h_{\mathrm{id}}=H\left(\mathrm{hk},\mathrm{id}\right)$. It outputs the secret key ${\mathrm{sk}}_{\mathrm{id}}$ as
  $$g^{1/(\beta +h_{\mathrm{id}})}.$$
- Aggregate(pk, ${\left\{\left({\mathrm{id}}_{i},sk_{i}\right)\right\}}_{i})\to \hat{\mathrm{sk}}$: The key aggregation algorithm computes the aggregated key as
  $$\hat{\mathrm{sk}}=\mathrm{DPP}\left({\left\{{\mathrm{sk}}_{i},x_{i}\right\}}_{i}\right)$$
  where $x_{i}=H\left(\mathrm{hk},{\mathrm{id}}_{i}\right)$.
- Enc$(m,\mathrm{mpk},\mathrm{id},1^{T})\to \mathrm{ct}$: The encryption algorithm takes a message *m*, a public key pk, an identity id and a bound *T* where *T* is the maximum number of aggregations. It randomly selects a random value $r\in Z_{p}$ and computes $h_{\mathrm{id}}=H\left(\mathrm{hk},\mathrm{id}\right)$. It, then, sets the following as the ciphertext ct:
  $$C_{i}:=g^{r\left(\beta +h_{\mathrm{id}}\right)\beta^{i}},C_{T}:=m\cdot e{\left(g,g\right)}^{r}$$
  It outputs ${\mathrm{ct}}_{\mathrm{id},0}:=({\left\{C_{i}\right\}}_{i=0}^{T-1},C_{T})$.
- AggDec($\hat{\mathrm{sk}},\mathrm{ct},\left({\mathrm{id}}_{1},\ldots ,{\mathrm{id}}_{\ell }\right),j\in \left[\ell \right])\to m$: The decryption algorithm takes the aggregated secret key $\hat{\mathrm{sk}}$ and the ciphertext ct and the identities $\left\{{\mathrm{id}}_{1},\ldots {\mathrm{id}}_{\ell }\right\}$. It parses ct as $\left({\left\{A_{i}\right\}}_{i=0}^{T-1},B\right)$ and computes the identity hash $x_{i}=H\left(\mathrm{hk},{\mathrm{id}}_{i}\right)$ for i∈[ℓ]\{j}. It then computes the coefficients $a_{i}\in Z_{p}$ for i ∈{0,…,ℓ−1} as in
  $$\prod_{i\in [\ell ]\backslash \{j\}}\left(y+x_{i}\right)=\sum_{i=0}^{\ell -1}a_{i}\cdot y^{i}\left(\mathrm{mod}\;\;\;p\right).$$
  It, then, outputs
  $$m=C_{T}\cdot e{\left(\prod_{i=0}^{\ell -1}{C_{i}}^{a_{i}},{\mathrm{sk}}_{\mathrm{id},\ell }\right)}^{-1}.$$

The correctness of the above scheme can be found in [15].
